# Supplementary material for: The Comprehensive Evaluation of Safflowers in Different Producing Areas by Combined Analysis of Color, Chemical Compounds, and Biological Activity
Source: Molecules. 2019 Sep 17;24(18):3381. doi: 10.3390/molecules24183381 (PMC6767200; doi:10.3390/molecules24183381)
Supplement: Supplementary file 1 [file molecules-24-03381-s001.pdf]

Table S1. Calibration curves, correlation coefficients, linearity ranges, LODs, and LOQs of the 16 compounds.

No	Compound	Calibration curves	r^2	Linear range	LOD (ng/mL)	LOQ (ng/mL)
**1**	hydroxysafflor yellow A	y = 75.889x - 129338	0.9998	33750~850000	2.060	8.240
**2**	safflomin C	y = 41.813x + 12896	0.9979	53.1001~27187.5	13.275	26.550
**3**	anhydrosafflor yellow B	y = 62.594x + 54690	0.9977	15.7166~64375	7.858	15.717
**4**	kaempferol	y = 0.9415x - 2327.9	0.9935	63.4766~32500	31.738	63.477
**5**	kaempferol-3-*O*-glucoside	y = 565.41x - 40147	0.9995	19.6457~40234.375	9.823	19.646
**6**	kaempferol-3-*O*-rutinoside	y = 601.05x + 144731	0.9993	9.4567~38734.375	1.182	4.728
**7**	kaempferol-3-*O*-β-sophoroside	y = 139.38x + 25489	0.9962	19.989~10234.375	4.997	9.995
**8**	6-hydroxykaempferol	y = 2.8269x - 6805.5	0.9963	917.9688~58750	14.343	97.969
**9**	6-hydroxykaempferol-3-*O*-β-D-glucoside	y = 267.82x - 59705	0.9983	124.5117~31875	62.256	124.512
**10**	6-hydroxykaempferol-3,6-di-*O*-β-D-glucoside	y = 179.93x + 64124	0.9987	29.2969~15000	14.648	29.297
**11**	6-hydroxykaempferol-3,6,7-tri-*O*-β-D-glucoside	y = 27.69x + 8860	0.999	65.3076~33437.5	32.654	65.308
**12**	quercetin	y = 36.144x - 11417	0.9993	122.0703~15625	7.629	15.259
**13**	rutin	y = 812.75x - 36966	0.9984	37.5367~9609.375	4.692	18.768
**14**	luteoloside	y = 812.75x - 36966	0.9984	37.5367~9609.375	2.346	18.768
**15**	apigenin	y = 57.555x - 42279	0.9947	42.1143~10781.25	2.632	42.114
**16**	quercetin-3-*O*-β-D-glucoside	y = 10.397x + 3514.9	0.9985	352.26~45089.281	176.130	352.260

Table S2. Precision, repeatability, stability, and recovery of 16 compounds.

Compund No.	Precision (RSD, %)		Repeatability (RSD, %, n = 6)	Stability	Recovery (%, n = 3)		
	**intraday**	interday			spike level (80%)	spike level (100%)	spike level (120%)
**1**	**1.17**	2.11	1.55	2.29	98.11(4.71)	104.62(3.61)	99.89(5.31)
**2**	1.35	2.88	1.79	2.98	103.65(3.45)	106.44(4.03)	102.30(3.57)
**3**	1.64	1.42	2.15	1.65	94.66(5.11)	104.71(3.55)	94.31(2.96)
**4**	2.08	2.53	2.67	2.43	97.23(4.54)	98.87(2.72)	96.19(4.28)
**5**	1.66	3.12	2.14	1.63	96.80(3.68)	103.57(3.44)	98.64(5.69)
**6**	3.87	1.65	2.39	2.44	97.34(4.02)	106.55(2.57)	104.11(3.35)
**7**	3.16	2.44	3.01	2.13	96.51(4.12)	105.34(1.96)	103.97(2.71)
**8**	4.56	3.83	3.22	3.11	99.30(5.19)	99.75(1.65)	98.21(4.33)
**9**	2.03	2.61	1.98	4.55	98.67(4.76)	103.73(2.37)	96.20(3.20)
**10**	3.89	5.02	3.76	3.60	97.68(3.81)	98.69(3.85)	97.26(3.62)
**11**	1.82	3.21	2.66	4.03	95.38(2.62)	99.31(4.97)	101.60(4.10)
**12**	3.94	2.88	3.55	3.54	98.83(1.69)	98.67(3.88)	103.55(1.37)
**13**	4.43	5.66	4.01	3.61	96.33(3.59)	103.22(1.39)	104.71(1.51)
**14**	2.31	2.87	2.39	2.84	94.89(4.79)	103.69(2.14)	96.40(3.76)
**15**	4.52	3.11	3.85	3.96	97.84(4.48)	96.97(1.44)	94.33(2.84)
**16**	3.34	4.02	3.06	2.37	96.46(3.97)	95.39(3.30)	96.67(4.35)

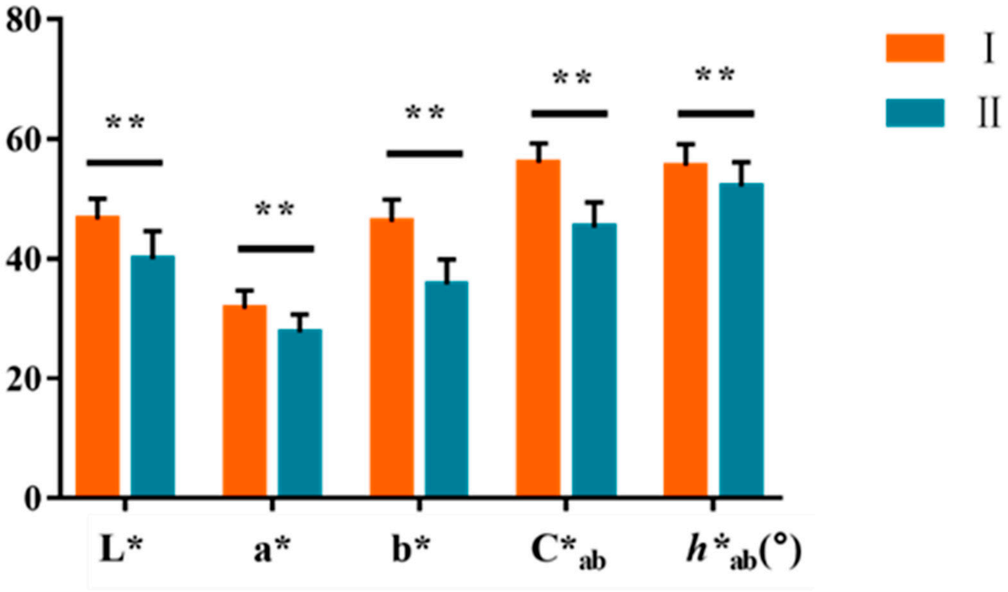

Figure S1. Comparison of colour indicators between class I and II of safflower.

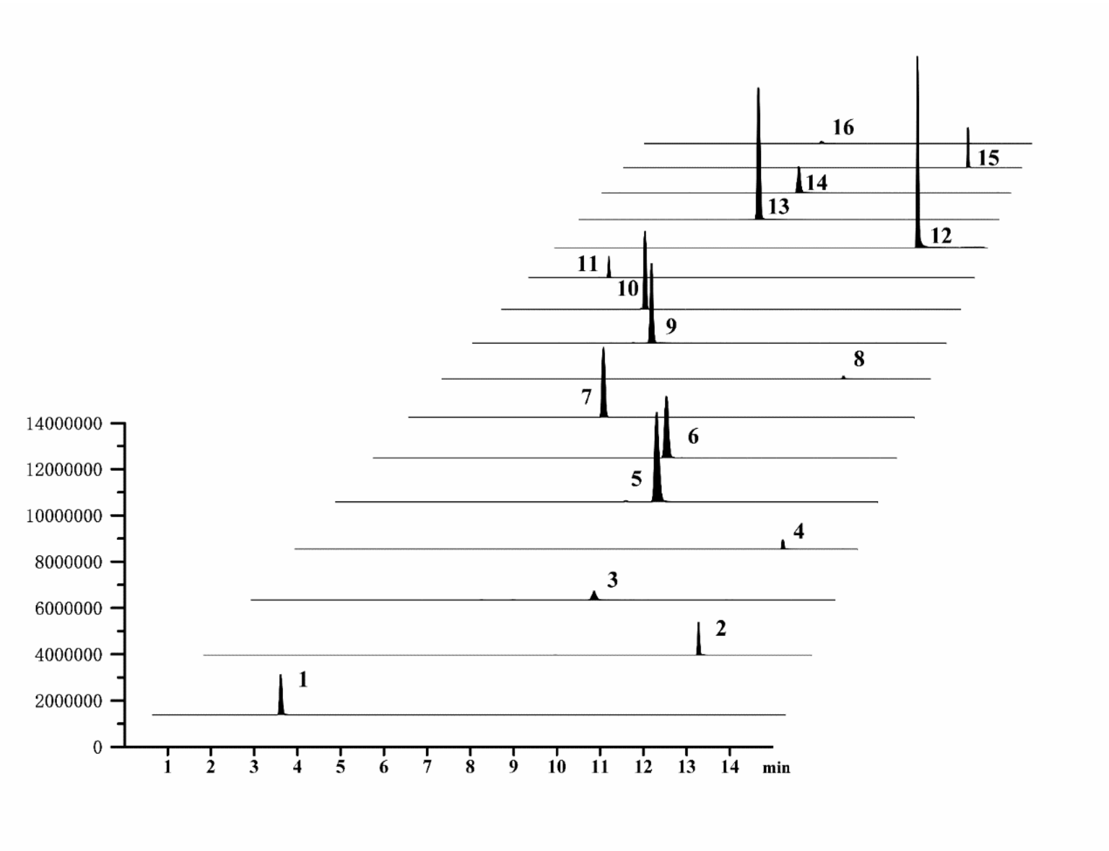

Figure S2. Representative chromatogram of 16 compounds in safflower.

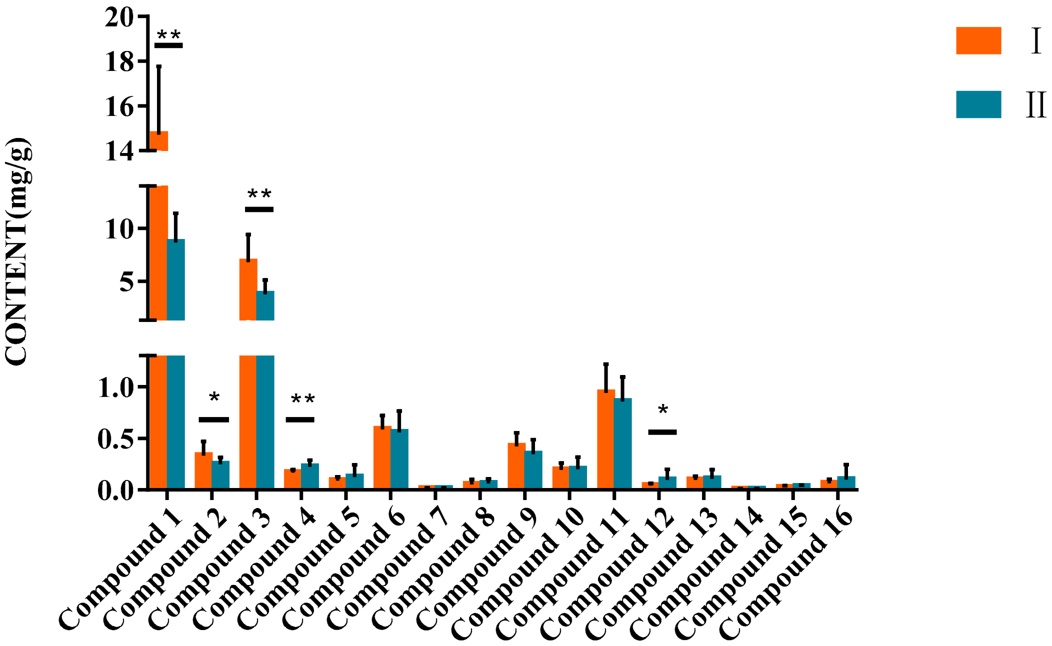

Figure S3. Quantitative determination of 16 compounds from class I and II of safflower.

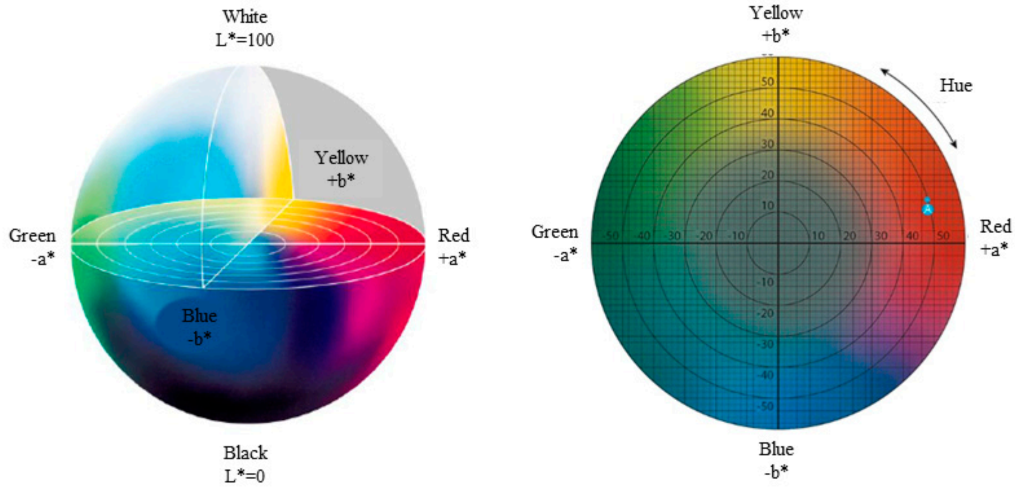

Figure S4. CIE LAB colour space.
